# Effect of Potato Pulp Pectic Polysaccharide on the Stability of Acidified Milk Drinks

**DOI:** 10.3390/molecules25235632

**Published:** 2020-11-30

**Authors:** Weixuan Sun, Wenhan Yang, Yuxue Zheng, Huiling Zhang, Haitian Fang, Donghong Liu, Xiangli Kong, Shiguo Chen, Xingqian Ye, Jinhu Tian

**Affiliations:** 1 National-Local Joint Engineering Laboratory of Intelligent Food Technology and Equipment, Zhejiang Key Laboratory for Agro-Food Processing, Integrated Research Base of Southern Fruit and Vegetable Preservation Technology, Zhejiang International Scientific and Technological Cooperation Base of Health Food Manufacturing and Quality Control, College of Biosystems Engineering and Food Science, Zhejiang University, Hangzhou 310058, China; weixuans357@163.com (W.S.); 21813075@zju.edu.cn (W.Y.); zhengyx0211@163.com (Y.Z.); dhliu@zju.edu.cn (D.L.); chenshiguo210@163.com (S.C.); 2 Ningxia Key Laboratory for Food Microbial-Applications Technology and Safety Control, Ningxia University, Yinchuan 750021, China; zhl5792@163.com (H.Z.); fanght@nxu.edu.cn (H.F.); 3 Ningbo Research Institute, Zhejiang University, Ningbo 315100, China; 4 Institute of Nuclear Agricultural Sciences, Zhejiang University, Hangzhou 310058, China; kongxiangli1980@hotmail.com

**Keywords:** potato pulp, acidified milk drinks, pectic polysaccharide, casein, stability

## Abstract

In order to broaden the application of potato pulp pectic polysaccharide (PPP) in stabilizing acidified milk drinks (AMDs) and investigate the stabilizing effect and physical properties of AMDs prepared with PPP, a comparative study was made among PPP, commercial high methoxyl pectin (HMP) and low methoxyl pectin (LMP). The zeta potential, rheology, particle size and serum separation of AMDs were evaluated after preparing with PPP, HMP and LMP, respectively. Results indicated that PPP led to lower serum separation than LMP (14.65% for AMDs prepared with 0.5% PPP compared to 25.05% for AMDs prepared with 0.5% LMP), but still higher than HMP (9.09% for AMDs prepared with 0.5% HMP). However, narrower particle size distribution and lower viscosity of AMDs was achieved by PPP than by LMP and HMP. PPP can electrostatically adsorb on the surface of casein and its abundant neutral sugar side chains would provide steric hindrance to prevent casein flocculation in AMDs. Our results might provide some new ideas for the application of PPP in improving the stability of AMDs.

## 1. Introduction

Pectin is a heteropolysaccharide that is mostly distributed in the primary cell walls of plants. The domains of pectic polysaccharide include homogalacturonan (HG), rhamnogalacturonan I (RG-I) and rhamnogalacturonan II (RG-II), where HG consists of a linear backbone, while RG-I and RG-II are highly ramified [1,2,3]. Normally, commercial HG-dominated pectin is derived from citrus peel or apple pomace and has a high GalA content (65%) [4,5]. Traditional HG-dominated pectin with more than 50% of methylation in GalA is termed as high methyl pectin (HMP), otherwise it is termed as low methyl pectin (LMP) [6].

In recent years, byproducts from other plants have also been considered to extract pectic polysaccharide [7,8]. It is worth noting that the monosaccharide composition of pectic polysaccharide from potato pulp is similar to that of traditional HG-dominated pectin, but the HG proportion and GalA content are quite different [6]. Different from citrus or apple derived pectin, potato pulp pectic polysaccharide (PPP) is branched, with a large amount of galactan (67% of RG-I) in the backbone [9]. Given its high galactan content, PPP is claim to have higher healthcare potential based on prebiotic performance and its action in the anti-cancer field (in vitro). For instance, potato RG-I pectic polysaccharide with abundant galactan side chains has high selective fermentability by probiotic strains (e.g., *Bifidobacterium* and *Lactobacillus*) [10,11]. On the other hand, β-galactose at the end of the abundant galactan side chains can bind to galectin-3 (a lectin associated with cancer), giving it higher anti-cancer potential than citrus or apple pectin [3,12]. 

In the past, PPP, with its highly branched structure, was largely unexploited due to its poor gelling properties [13]. However, the large amount of galactan side chains and high degree of acetylation endow PPP with a good emulsifying ability and it might potentially be useful in the food industry. Yang et al. revealed that the acetyl groups confer amphiphilic characteristics on PPP, which enable it to exist on the water–oil interface, thus showing better emulsifying stability than citrus- and apple-derived pectin [6]. Khodaei et al. emphasized the advantage of the multi-side chain structure on the high emulsifying stability of PPP [14]. 

Acidified milk drinks (AMDs) are popular dairy beverages produced by adding acidulant to milk or fermenting with *Lactobacillus*. Casein is the main constituent of milk protein (80%) and characterised with flexible conformation without specific secondary structure [15]. Four sub-fractions (αs1-, αs2-, β- and κ-casein) bond together via hydrophobic interactions and through the bridging of colloidal calcium phosphate (CaP) to form a casein micelle [16]. Under the normal pH of milk (pH 6.7), the hydrophilic chains of κ-casein would protrude from the surface of casein micelles and form a hairy outer layer on casein micelles, providing steric hindrance among casein micelles to make them disperse stably [17]. The dissociation and rearrangement of the four sub-fractions of casein is rather complex during milk acidification, while it was normally recognized that the collapse of the extended conformation and the dissociation of κ-casein was the main reason for the destabilization of casein micelles [18].

Introducing anionic polysaccharides as stabilizers can avoid casein aggregation in AMDs effectively, because the anionic polysaccharides could adsorb on the casein surface and help restore the stabilization of casein [18]. Commercial HG-dominated pectin is commonly used in the stabilization of AMDs [19]. When pectin is involved in preparing AMDs, the negatively charged carboxyl groups in GalA residues are considered to electrostatically bind to the positively charged patches on the casein micelle surface, whereas non-adsorbing parts of the sugar chains would protrude as loops and tails around the casein micelles, providing steric hindrance against casein aggregation [19]. Normally, HMP is more widely used than LMP—on the one hand, HMP has low electrostatic affinity for the casein micelle surface due to the lower content of free carboxyl, on the other, HMP can be less calcium-sensitive than LMP [18,20]. Numerous studies have indicated that inter-chain cross-linking of pectin induced by calcium bridging can hinder electrostatic association of the pectin with casein [21]. 

The RG-I-dominated PPP contains fewer GalA, so it has lower charge density and higher resistance to calcium bridge cross-linking like HMP [22]. In addition, the rich neutral sugar side chains on PPP have a similar effect as the low-affinity blocks of HMP. Peterson et al. found that the RG-I structure in PPP enables the distribution of negative charges of GalA residues along the backbone, and the entropy-enriched galactan side chains would protrude from the casein surface and contribute more steric hindrance for casein micelles [23]. However, their study was conducted at a pH of 5.5, and whether PPP can be a stabilizer in AMDs (pH 3.6–4.6, lower than the isoelectric point of casein) has not yet been reported [24]. 

Considering the low cost and high health potential, the main objective of present study was to investigate the behaviour of PPP in the stabilization of AMDs. Based on the structural characteristics and the previous work of PPP, reasonable hypothesis is that PPP can be a good AMDs stabilizer. In this investigation, the stabilizing ability of PPP was evaluated by comparing the difference in the stability and physical properties of AMDs prepared with PPP and commercial citrus pectin (HMP and LMP). Our study might broaden the applications of PPP in the food industry and provide a potential optional stabilizer for AMDs.

## 2. Materials and Methods

### 2.1. Materials and Reagents

Dried potato pulp was generously provided by Jialiyuan Co., Ltd. (Ningxia, China). Powdered skimmed milk was obtained from Fonterra Co., Ltd. (Wellington, New Zealand). Commercial HMP and LMP (from citrus peels, LMP were from the de-esterification of HMP with alkaline) were bought from Andre Pectin Co., Ltd. (Yantai, China). Standard monosaccharides including fucose (Fuc), rhamnose (Rha), arabinose (Ara), galactose (Gal), glucose (Glc), mannose (Man), xylose (Xyl), galacturonic acid (GalA) were purchased from Aladdin Chemical Co., Ltd. (Shanghai, China). Other chemicals and reagents were all analytical grade and purchased from Sinopharm Chemical Reagent Co., Ltd. (Shanghai, China).

### 2.2. Extraction of Potato Pulp Pectic Polysaccharide

Dried potato pulp was ground and sieved through a 60-mesh sieve. α-amylase and amyloglucosidase were applied to remove the residual starch [9]. Destarched potato pulp was dispersed in 5% citric acid monohydrate (at around pH 1.94, 1:15 solid to liquid ratio) and the pH of the mixture was adjusted to 2 with 10% (*w*/*v*) citric acid monohydrate or 0.1 mol/L NaOH. Then, the mixture was heated at 88 °C for 1 h and filtered through a G3 sintered glass filter. The recovered filtrate was neutralized and absolute ethanol was added to the filtrate until a final ethanol concentration of 40% was achieved. After being kept at 4 °C for 12 h, the precipitate was collected by centrifugation at 5095× *g* for 10 min and washed twice with ethanol. The precipitates were re-dissolved in distilled water, dialyzed (8–14 kDa) for 2 days and freeze-dried to obtain the potato pulp pectic polysaccharide (PPP).

### 2.3. Protein, Uronic Acid Content and Monosaccharide Composition

Protein content in PPP, HMP and LMP was measured with a Bradford Protein Assay Kit (Beyotime Biotechnology, Shanghai, China). Determination of uronic acid content was referred to Filisetti-Cozzi and Carpita [25]. Briefly, galacturonic acid at different concentrations (0–0.1 mg/mL) was used as standard. Each sample (PPP, HMP and LMP) was completely dissolved and prepared as a 0.1 mg/mL solution. Then, sulphamate/*m*-hydroxydiphenyl assay was applied to determine uronic acid content. Monosaccharide composition was measured with a high-performance anion exchange chromatography (HPAEC) (ICS-5000+, Thermo Fisher Scientific, Fremont, CA, USA) according to Zhang at al. [26]. Briefly, 2.5 mg PPP, HMP and LMP were accurately weighed, dissolved in 2 mL trifluoroacetic acid (2 mol/L) and hydrolysed at 110 °C for 8 h, respectively. The hydrolysates were dried using a steam of high-purity nitrogen and dissolved in 10 mL deionized water. Then the solutions were filtered through a 0.22 μm membrane prior to analysis. The chromatographic column used for analysis included a CarboPac guard column (4 × 50 mm, Dionex, Thermo Fisher Scientific) and a CarboPac PA1 analytical column (4 × 250 mm, Dionex, Thermo Fisher Scientific). The mobile phases were deionized water (solvent A), 200 mM NaOH (solvent B) and 100 mM NaOAc (solvent C). Elution was initiated at 88% A and 12% B for 15 min, followed by 100% C for the following 20 min. Different concentrations (0.02, 0.04, 0.06, 0.08, 0.1, 0.2 mmol/L) of monosaccharides mixtures were used as standards for calibration.

### 2.4. Molecular Weight Determination

The molecular weight of PPP, HMP and LMP was analysed by high-performance size-exclusion chromatography (HPSEC, Shimadzu, Tokyo, Japan), which consisted of an Optilab rEX differential refractometer (RI, Wyatt Technology, Santa Barbara, CA, USA) and a DAWN HELEOS II multi-angle light scattering detector (MALLS, Wyatt Technology). Briefly, isocratic elution with 0.15 mol/L NaCl containing 0.02% NaN_3_ at a flow rate of 0.5 mL/min was performed on the Shodex SB-804 HQ column and the Shodex SB-806 HQ column. A dn/dc value of 0.138 mL/g was used for the molecular weight analysis. The samples were dissolved in the mobile phase at a concentration of 2 mg/mL. After filtering through 0.22 μm membrane, 25 μL solution was injected through a sample loop. Weight average molecular weight (Mw) was analysed using ASTRA 7.1.2.5 software (Wyatt Technology). 

### 2.5. Degree of Esterification Measurement

The degree of methyl esterification (DM) and degree of acetylation (DA) of PPP, HMP and LMP were determined by a ^1^H-NMR method [27]. To be specific, 10 mg of each sample was dissolved in 1 mL of 0.4 M NaOH in D_2_O and incubated at room temperature for 2 h. The supernatant was collected by centrifugation at 3000× *g* for 30 min. Then 100 μL of the sodium trimethylsilyl propionate (TSP) internal standard (2 mg/mL in D_2_O) was added to the supernatant. The mixtures were filtered through a 0.45 μm membrane prior to transfer to NMR tubes. Each sample was scanned 64 times and the ^1^H-NMR spectra were collected by a DD2-600 MHz spectrometer (Bruker, Hamburg, Germany) at a sweep width of 20.03 ppm and acquisition time of 2.73 s (Supplementary Materials). The content of methanol and acetic acid was determined by manual integration of the peaks of methanol (3.36 ppm), acetic acid (1.92 ppm) and TSP (0 ppm) and the values of DM and DA were calculated according to the following Equations (1)–(4), where A(x) was the spectral area of the analyte, M(x) was the relative molecular mass of the analyte, mol of GalA was calculated according to the uronic acid content determined by sulphamate/*m*-hydroxydiphenyl assay [27]:

(1) $$\mathrm{mol}\;\mathrm{of}\;\mathrm{methanol}=\frac{A\left(\mathrm{Methanol}\right)\cdot \mathrm{mg}(\mathrm{TSP})}{A\left(\mathrm{TSP}\right)\cdot M(\mathrm{TSP})}$$

(2) $$\mathrm{mol}\;\mathrm{of}\;\mathrm{acetic}\;\mathrm{acid}=\frac{A\left(\mathrm{Acetic}\;\mathrm{acid}\right)\cdot \mathrm{mg}(\mathrm{TSP})}{A\left(\mathrm{TSP}\right)\cdot M(\mathrm{TSP})}$$

(3) $$\mathrm{DM}=\frac{\mathrm{mol}\;\mathrm{of}\;\mathrm{methanol}}{\mathrm{mol}\;\mathrm{of}\;\mathrm{GalA}}\;\times \;100\%$$

(4) $$\mathrm{DA}=\frac{\mathrm{mol}\;\mathrm{of}\;\mathrm{acetic}\;\mathrm{acid}}{\mathrm{mol}\;\mathrm{of}\;\mathrm{GalA}}\;\times \;100\%$$

### 2.6. Preparation of AMDs

Preparation of AMDs samples was based on the method described by Peterson et al., with some modifications [23]. PPP, HMP and LMP were dissolved in deionized water at a certain content and stirred at 75 °C for 30 min to obtain stabilized solutions. Powdered skimmed milk was reconstituted to 17% (*w*/*v*) non-fat milk drink using deionized water with 0.02% (*w*/*v*) sodium azide to prevent microbial growth. The non-fat milk drink was stirred at 60 °C for 1 h to hydrate the milk protein and then 1% (*w*/*v*) glucono-δ-lactone was added as the acidulant. After acidification for 16 h, certain amounts of PPP, HMP and LMP were mixed with AMDs to obtain a final milk powder content of 8.5% (*w*/*v*) and to achieve concentrations ranging from 0.0% (*w*/*v*) to 0.5% (*w*/*v*) pectic polysaccharide stabilizers in the final AMDs. The pH of the final AMDs was adjusted to 4 with 10% citric acid, and underwent homogenization at 40 MPa through an ultra-high-pressure homogenizer (NanoGenizer, Genizer, Irvine, CA, USA). 

### 2.7. Zeta Potential

The zeta potential was determined by a Zetasizer Nano-ZS (Malvern Instruments, Worcestershire, UK), according to Zhao, Qi, Liu, Zeng, and Yang [28]. The zeta potential value was collected based on laser doppler velocimetry. All the testing AMDs (overnight after preparation) were diluted 100-fold in citrate buffer (pH = 4) before adding in the sample pool. Zeta potential of 0.1% (*w*/*v*) pectic polysaccharide (PPP, LMP and HMP) solution in pH 2–7 were also quantified. The equilibration time is 120 s. All the samples were measured in triplicate and the data were reported as averages. 

### 2.8. Particle Size

Particle size of the AMDs with PPP, HMP and LMP was measured using a LS-320 Particle Size Analyser (Beckman Coulter, Brea, CA, USA). All the measurements were performed two days after preparation. The refractive indices of particle and solvent were chosen as 1.50 and 1.33, respectively [5]. The AMDs samples were dripped into degassed ultrapure water and the particle size was measured until obscurity ranged from 7 to 12.

### 2.9. Rheology

The apparent viscosity of AMDs (stabilized by 0.5% PPP, HMP or LMP), serum (obtained by 3000× *g* centrifugation of AMDs) and stabilizer solutions (0.5%) were measured with a HAAKE RheoStress 6000 rheometer (Thermo Scientific, Fremont, CA, USA). A frequency sweep from 0.1 to 10 Hz was performed and the constant shear strain of 1 was applied to investigate the effect of different stabilizers (0.5%) on the viscoelasticity of AMDs according to Jensen at al. [5]. Rheological analyses were performed two days after preparation; only samples without visible sedimentation were tested.

### 2.10. Serum Separation

Serum separation of AMDs was measured to evaluate the drink stability. AMDs (10 mL) were placed in measuring tubes and stored at 4 °C for 7 days. Serum separation was calculated using the following Equation (5) [29]:

(5) $$\mathrm{Serum}\;\mathrm{separation}\;(\%)\;=\frac{\mathrm{Separated}\;\mathrm{serum}\;\mathrm{phase}\;(\mathrm{mL})\;}{\mathrm{Freshly}\;\mathrm{prepared}\;\mathrm{AMDs}\;(\mathrm{mL})}$$

### 2.11. Confocal Laser Scanning Microscopy

The microstructural differences of flocculates in AMDs were observed according to Tromp et al. [18]. Briefly, 100 μL fluorescein-5-isothiocyanate (FTIC, 2 mg/mL) was added into 5 mL shaken AMDs with different stabilizers (blank, 0.5% PPP, 0.5% HMP and 0.5% LMP) and kept for 7 days. FTIC-labelled milk protein dispersion (10 μL) was put on a glass slide and covered with a cover slip. Observation was carried out on a Leica TCS SP confocal scanning light microscope (Leica Microsystems Inc., Heidelberg, Germany) with excitation and emission wavelengths of 500 and 530 nm, respectively.

### 2.12. Statistical Analysis

The data were reported as mean ± standard deviation. One-way analysis of variance was conducted by Tukey’s test using SSPS 17.0 software (IBM Inc., New York, NY, USA); differences were considered significant at *p* < 0.05 level.

## 3. Results and Discussion

### 3.1. Compositional Characterization of PPP, HMP and LMP

The compositional properties. which include protein and uronic acid content, degree of methylation and acetylation (DM and DM) as well as molecular weight (Mw), are listed in Table 1. As shown, compared to HMP and LMP, PPP had the lowest uronic acid content. This was in accordance with previous studies where the uronic acid content of potato derived pectic polysaccharide was much lower than that from other sources (i.e., citrus peel, apple pomace and grape peel, etc.) [9,30]. PPP also showed a much lower DM than HMP and LMP, indicating that GalA in PPP was less methyl-esterified. However, highest DA was obtained by PPP, which was consistent with previous studies [6,23]. PPP exhibited the highest molecular weight among the three pectic polysaccharides, which could be attributed to the highly branched structure [2,31].

The molar ratio of eight monosaccharides quantified by HPAEC is illustrated in Table 2. The results showed that monosaccharides in the three pectic polysaccharides are mainly Rha, Ara, Gal, Glc and GalA, while Fuc, Man and Xyl account for low proportions. Molar ratio of each monosaccharide was quite different for PPP HMP and LMP. Most notably, GalA was the majority in HMP (64.37 mol%) and LMP (52.03 mol%), while the molar ratio of GalA was only 34.74 mol% in PPP and Gal was the highest proportion (36.08 mol%). This might be explained by the abundant galactan side chains in the RG-I domain PPP [6,9]. LMP was obtained through de-esterification of HMP under weak alkaline conditions, which possibly lead to the hydrolysis of the HG structure, thus the molar ratio of GalA in LMP was lower (*p* < 0.05) than that of HMP. 

### 3.2. Zeta Potential

**Figure 1:**
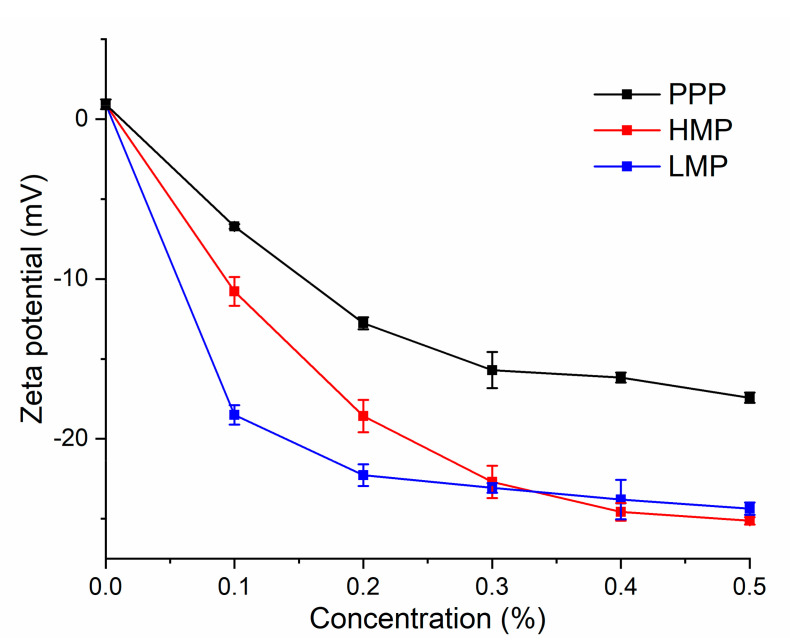
Zeta potential of AMDs as a function of stabilizers concentration (from 0 to 0.5%).

Zeta potential can describe the magnitude of electrical charge on particles and has been widely used in dairy research as an indicator of the surface charge of colloidal casein [28]. The zeta potential of AMDs prepared with PPP, HMP and LMP at various concentrations are shown in Figure 1. As it was below the isoelectric point of casein, AMDs prepared without any stabilizers was positively charged. PPP, HMP and LMP were negatively charged under pH 4 (Figure S1) and the zeta potential of AMDs turned to negative when stabilized by the three pectic polysaccharides, which indicated that casein micelles get covered by the anionic polysaccharide chains. Meanwhile, the zeta potential of AMDs showed decreasing trend as the concentration of the pectic polysaccharide increased. AMDs prepared with PPP (from 0.1 to 0.5%) showed a zeta potential ranged from −6.71 to −17.43 mV, generally higher than that with HMP (from −10.77 to −25.13 mV) and LMP (from −18.50 to −24.37 mV). This result indicated that the electrostatic repulsive forces of casein colloidal covered with PPP was weaker than those covered with HMP and LMP. PPP had lower content of GalA and thus a lower negative charge density (Figure S1); therefore, PPP exhibited weaker electrostatic adsorption with a surface of positively charged casein. The lowest zeta potential was observed for AMDs prepared with LMP when the concentration of pectic polysaccharides was lower than 0.3%. However, at high concentration (>0.4%), HMP led to a slightly lower zeta potential (*p* < 0.05) than that for LMP. This was presumably caused by the higher calcium sensitivity of LMP, similar results were also reported by Everett and McLeod [32].

### 3.3. Particle Size

**Figure 2:**
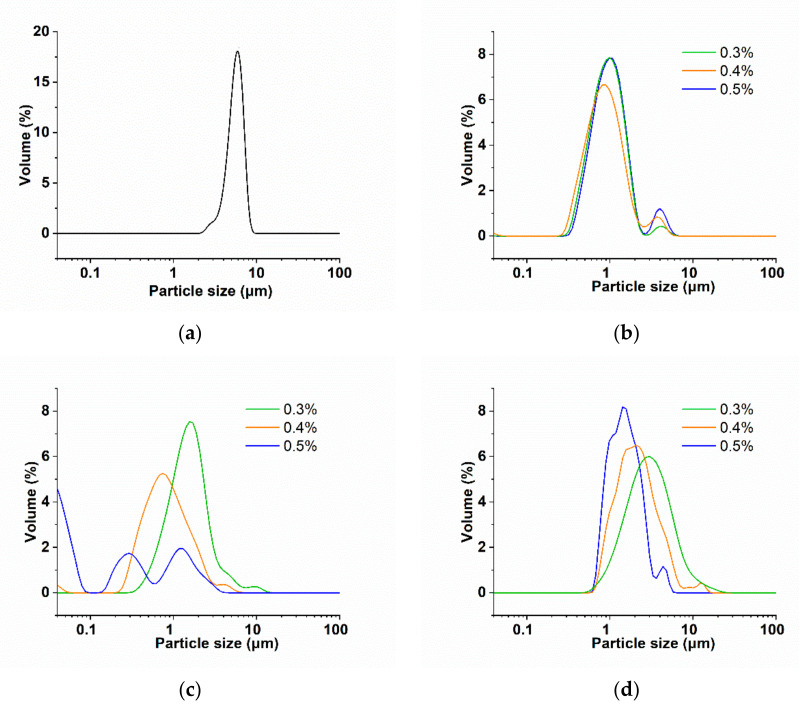
Particle size distribution of AMDs prepared without any stabilizers (**a**) and AMDs prepared with PPP (**b**), HMP (**c**) and LMP (**d**) at concentrations of 0.3%, 0.4% and 0.5%.

The particle size distribution of AMDs prepared with PPP, HMP and LMP was measured after storage for two days. As shown in Figure 2a, a monomodal distribution with particle sizes ranging from 2 to 10 μm was observed for the blank, which indicated the wide aggregation of casein micelles without any stabilizers [5]. After adding PPP (0.3%, 0.4% and 0.5%), the volume peak shifted from monomodal to bimodal (the major peak ranged from 0.25 to 2 μm and the small peak was around 4 μm) (Figure 2b). It was worth noting that the height of the small peak around 4 μm was positively associated with the concentration of PPP, which might be explained by the self-aggregation of PPP [23]. It seemed that 0.3 to 0.5% PPP did not affect the particle size distribution of the AMDs, which might suggest that 0.3% PPP was sufficient to cover all the casein, while the non-adsorbing PPP in serum could hardly contribute to decreasing the size of casein particles.

Certain amounts of HMP and LMP (i.e., 0.3% for HMP and 0.3% and 0.4% for LMP) resulted in broad peaks with tails extending to larger diameters than the blank (>10 μm) (Figure 2c,d), indicating promoted casein aggregation due to bridging flocculation. When pectin stabilizers were not enough to cover all the casein, two or more casein micelles would interconnect and shared one polysaccharide chain, which was recognized as a bridging effect [20,33]. Bridging flocculation was not observed when using PPP (0.3–0.5%) as stabilizer. Electrostatic interaction between PPP and casein was much weaker due to the low charge density of PPP, which may be the reason that PPP rarely form a bridge to electrostatically connect adjacent casein micelles. 

Among the different AMDs, the peak of the smallest size was observed with 0.5% HMP. However, the particle size distribution was broad with three volume peaks appearing, indicating that the particle size of casein micelles in AMDs with HMP varied greatly.

### 3.4. Rheological Analysis

**Figure 3:**
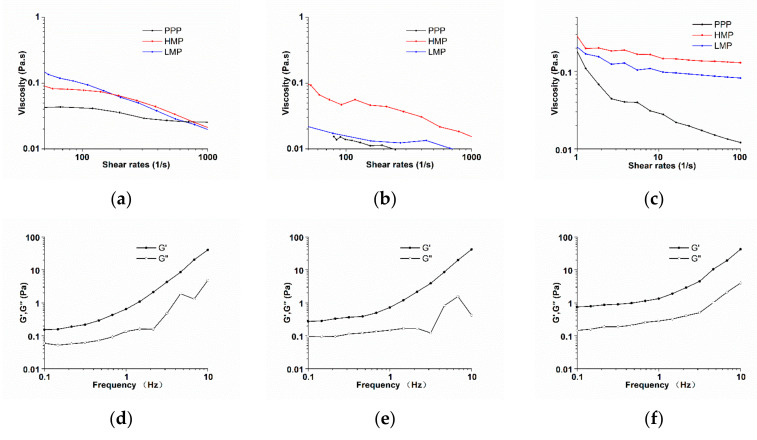
Steady shear flow curves: (**a**) of AMDs prepared with 0.5% different pectic polysaccharides, (**b**) serum of AMDs prepared with 0.5% different stabilizers, (**c**) solution of 0.5% PPP, HMP, LMP; The frequency sweep (0.1–10 Hz) of AMDs prepared with 0.5% different stabilizers: (**d**) PPP, (**e**) HMP, (**f**) LMP.

The apparent viscosity of AMDs prepared with PPP, LMP and HMP is shown in Figure 3a. The shear thinning behaviour of AMDs, which was induced by the deformation and destruction of casein flocculates under increased shear rate, was negatively associated with the stability of milk drinks [29]. For a stable AMDs system, the apparent viscosity would no longer change with increasing shear rate, which showed as a Newtonian flow behavior [29]. As shown in Figure 3a, Newtonian flow character started to develop in the PPP-stabilized AMDs at high shear rates (>200 s^−1^), suggesting less flocculates in it. LMP gave the highest viscosity of AMDs at low shear rates (<140 s^−1^). However, the apparent viscosity decreased sharply as the shear rate increased, indicating the widely occurring flocculation and aggregation in AMDs. Tromp found that up to 90% pectin could not electrostatically adsorb on the casein micelle surface, while the non-adsorbed pectin would develop into a viscous network, contributing to the increasing of AMDs’ viscosity and restrict the suspended casein particles from settling [18]. HMP led to a markedly higher serum viscosity (Figure 3b), indicating a more significant thickening effect of the non-adsorbed HMP on the AMDs. As shown in the solution viscosity (Figure 3c), LMP itself demonstrated higher viscosity than HMP. However, the viscosity of serum with non-adsorbed LMP was much lower (Figure 3b). Non-adsorbed LMP in serum is prone to be calcium cross-linked and formed gel which could largely be deposited by centrifugation [32,34], therefore, low apparent viscosity was observed in this result. PPP had the lowest solution viscosity (Figure 3c), which might attribute to the lowest GalA content, and it also led to the lowest serum viscosity (Figure 3b), suggesting that non-adsorbed PPP had a limited thickening effect on AMDs.

The viscoelasticity analysis showed that the storage modulus (G′) was higher than the loss modulus (G″) under the frequencies from 0.1 to 10 Hz for all the samples, indicating a gel-like structure (Figure 3d–f) [5,28]. Both G′ and G″ were higher in AMDs with LMP, suggested that the gel network was much stronger for AMDs prepared with LMP than PPP and HMP. And this might attribute to the presence of casein flocculates as well as LMP self-cross-linked gel [29]. 

### 3.5. Serum Separation

**Figure 4:**
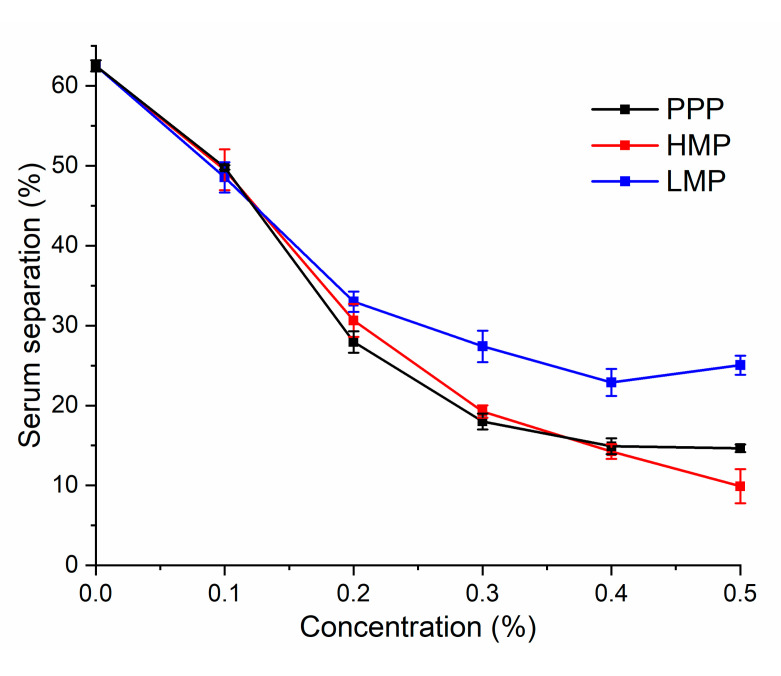
The percentage of serum separation of AMDs after storage for 7 days as a function of stabilizers concentration (from 0 to 0.5%).

The stability of AMDs prepared with HMP, LMP and PPP of various concentrations was also evaluated by determining serum separation at room temperature after storage for seven days (Figure 4). 

The highest serum separation (62.5%) occurred in AMDs prepared without any stabilizer. PPP and HMP resulted in a much lower amount of serum separation than that of LMP, when the stabilizer concentration was lower than 0.4%, no significant difference was observed between PPP and HMP. Since the increased amount of non-adsorbed PPP could hardly thickened the aqueous phase and reduce casein sedimentation, a high concentration of PPP (0.5%) did not cause further reduction of serum sedimentation as was seen with HMP. The amount of serum separation reduced when the concentration of LMP was increased from 0.1 to 0.4%; however, 0.5% LMP caused a higher serum separation than that of 0.4% LMP. This might attribute to depletion flocculation—the gel developed by cross-linking of excess LMP in serum was thermodynamically incompatible with casein collodials [33].

### 3.6. Confocal Laser Scanning Microscopy

**Figure 5:**
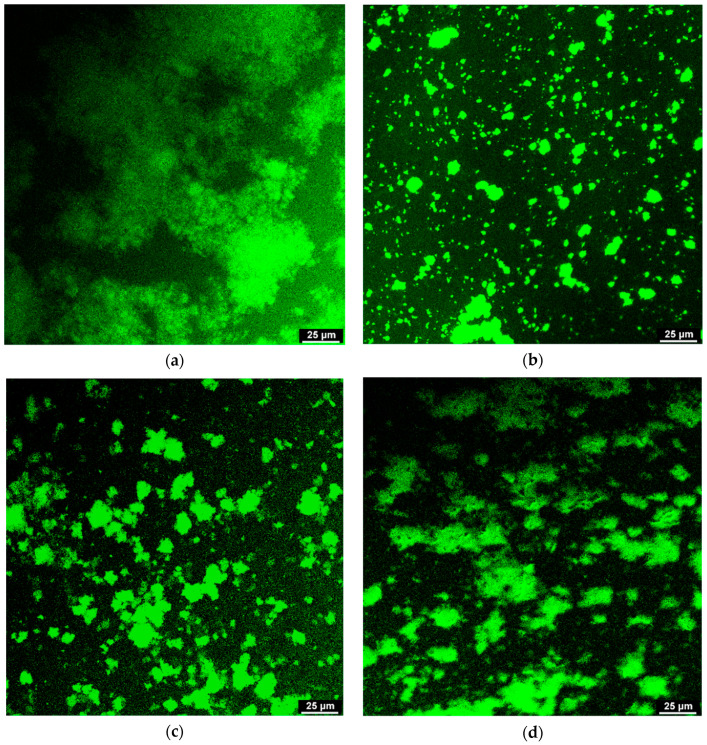
CLSM images of casein flocculates in AMDs prepared without any stabilizers (**a**), AMDs stabilized by PPP (**b**), HMP (**c**) and LMP (**d**) at 0.5% concentration after storage for 7 days.

Confocal laser scanning microscopy (CLSM) was applied to observe the microstructural difference of flocculates in AMDs after storage for seven days. As shown in Figure 5a, the casein flocculates in the blank were interconnected and distributed over a large area, indicating the formation of a macroscopic casein network [24]. 

Droplet-like flocculates of the smallest size (diameter < 15 μm) were observed in the AMDs prepared with 0.5% PPP (Figure 5b), indicating that PPP inhibited the aggregation of casein micelles effectively. Flocculates in the AMDs stabilized by 0.5% HMP were generally larger than those of AMDs stabilized with PPP (Figure 5c). However, less serum separation was observed for HMP (as indicated in the serum separation results), which was perhaps due to the thickening effect of non-adsorbing HMP, which prevented sedimentation of the casein flocculates. In addition, HMP led to a loose casein flocculates structure [35]. Compared with HMP and PPP, 0.5% LMP-stabilized AMDs had the largest flocculation particle size (d > 25 μm). This result was in accordance with the higher serum separation percentage. The casein flocculates showed an irregular block shape (Figure 5d), which might indicate the occurrence of depletion flocculation induced by LMP.

## 4. Conclusions

A comparative study was performed to verify the possibility of PPP as an AMDs stabilizer. Under pH 4, PPP could adsorb on the casein surface, reducing casein flocculation and sedimentation in AMDs. As for the stabilizing effect, PPP resulted in much smaller particle size than LMP and narrower particle size distribution than HMP and could effectively maintain the dispersion of casein after seven days’ storage. Serum separation in AMDs prepared with PPP was lower than that of LMP, but slightly higher than that of HMP. The different structural characteristics of PPP, especially its low HG proportion and rich galactan side chain, accounted for difference in the stability and physical properties of AMDs. In general, PPP can be considered as a potential non-thickening AMDs stabilizer, which can better maintain the fluidity of AMDs. 

## Figures and Tables

**Table 1:** Compositional properties of PPP, commercial HMP and LMP ^1^.

Property	PPP	HMP	LMP
Protein (%)	1.31 ± 0.30 ^a^	1.25 ± 0.34 ^a^	0.70 ± 0.16 ^b^
Uronic acid content (%)	34.02 ± 4.30 ^c^	69.18 ± 5.56 ^a^	60.11 ± 1.92 ^b^
DM (%)	8.32	66.29	30.14
DA (%)	11.33	3.85	1.56
Mw (kDa)	420.9	234.6	187.0

^1^ Different lowercase letters in the same row indicate a statistically significant difference (*p* < 0.05).

**Table 2:** Molar ratio of monosaccharides in PPP, HMP and LMP ^1^.

	Monosaccharide Composition (mol%)							
	Fuc	Rha	Ara	Gal	Glc	Man	Xyl	GalA
PPP	1.38 ± 0.02 ^a^	13.32 ± 0.16 ^a^	8.49 ± 0.10 ^b^	36.08 ± 0.60 ^a^	3.23 ± 0.07 ^c^	1.33 ± 0.03 ^a^	1.43 ± 0.04 ^a^	34.74 ± 0.80 ^c^
HMP	0.74 ± 0.04 ^c^	6.19 ± 0.71 ^c^	9.68 ± 1.07 ^b^	12.95 ± 1.09 ^b^	5.34 ± 0.46 ^b^	0.30 ± 0.15 ^b^	0.43 ± 0.11 ^b^	64.37 ± 3.11 ^a^
LMP	1.13 ± 0.09 ^b^	7.76 ± 0.14 ^b^	20.40 ± 0.34 ^a^	12.04 ± 0.41 ^b^	6.47 ± 1.40 ^a^	0.04 ± 0.04 ^c^	0.12 ± 0.02 ^c^	52.03 ± 1.40 ^b^

^1^ Different lowercase letters in the same row indicate a statistically significant difference (*p* < 0.05).

## Supplementary Materials

The following are available online. Figure S1: Zeta potential of PPP, HMP and LMP (0.1%, *w*/*v*) as a function of pH.
