# Supplementary figures and images for: Effect of Potato Pulp Pectic Polysaccharide on the Stability of Acidified Milk Drinks
Source: Molecules. 2020 Nov 30;25(23):5632. doi: 10.3390/molecules25235632 (PMC7731407; doi:10.3390/molecules25235632)

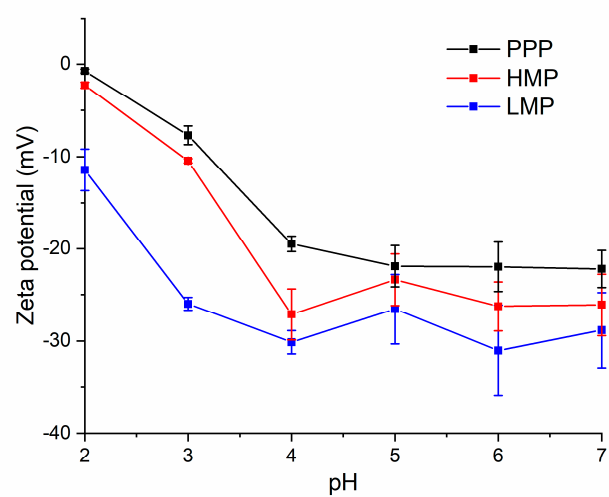

**Figure S1.** Zeta potential of PPP, HMP and LMP (0.1%, w/v) as a function of pH.

Supplement: Supplementary file 1 [file molecules-25-05632-s001.pdf]
